# Supplementary material for: Using natural history collections to investigate changes in pangolin (Pholidota: Manidae) geographic ranges through time
Source: PeerJ. 2021 Feb 11;9:e10843. doi: 10.7717/peerj.10843 (PMC7882139; doi:10.7717/peerj.10843)
Supplement: Supplemental Information 1 [file peerj-09-10843-s001.pdf]

Supplementary Materials from "*Using natural history collections to investigate changes in pangolin (Pholidota: Manidae) geographic ranges through time.*"

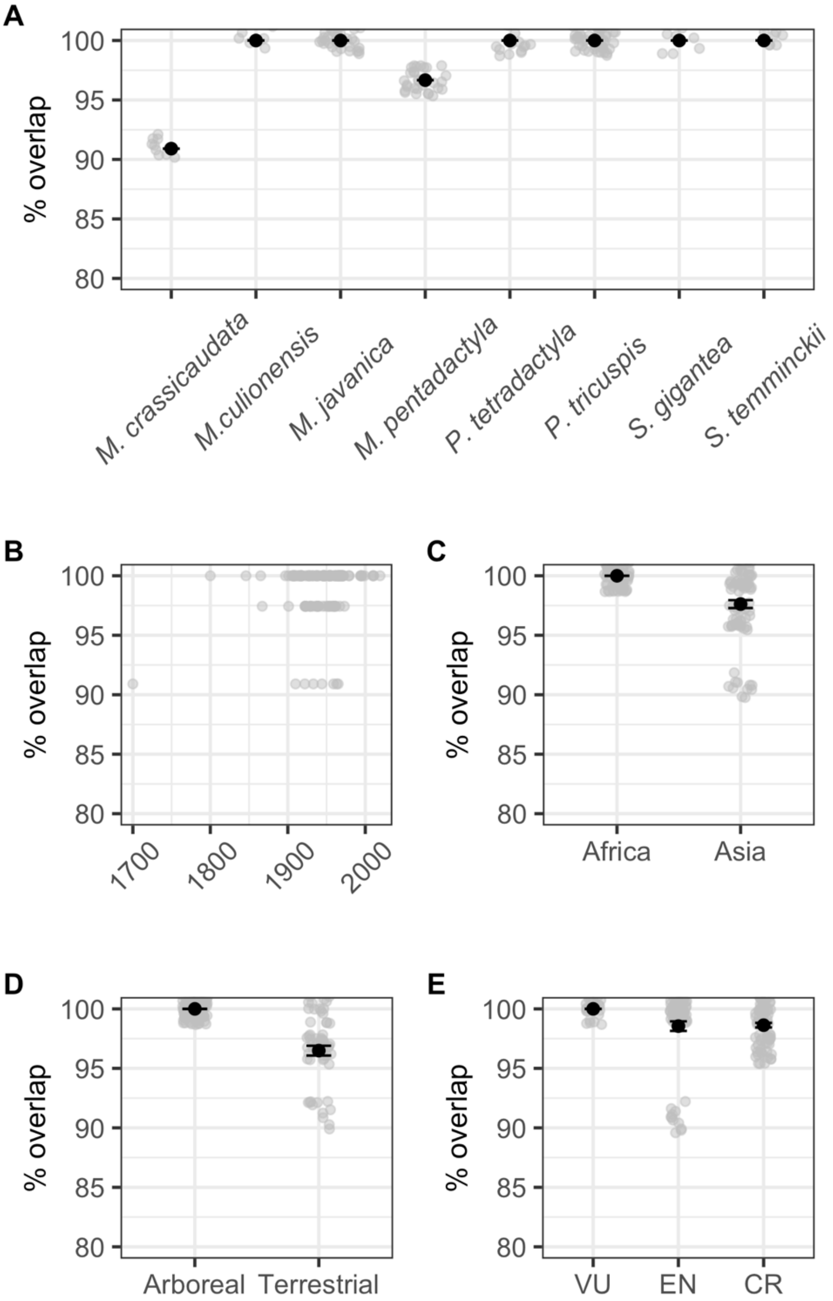

Figure S1: Correlates of percentage locality overlaps for each species, for specimens with certainty scores ≥ 50%, extents < 50km and excluding duplicates. (A) Species. (B) Collection year. (C) Continent. (D) Ecology. (E) IUCN Red List status. Black points are means, error bars are standard errors, grey points are the raw data.

Table S1: Specimens excluded from our analyses after georeferencing. We excluded zoo animals, specimens recorded from ports rather than collection localities, and specimens with localities more than 100km from the current species range and not unambiguously within the range of another closely related species. This does not include specimens with extents > 1000km as these are automatically removed from the analyses.

Specimen	Species	Justification
NHMUK_27.12.1.203	*Manis crassicaudata*	Outside of current range but inside the range of *M.pentadactyla* and *M.javanica*. Unclear if this is a taxonomic error.
NHMUK_76.139	*Manis crassicaudata*	Zoo animal (Sri Lanka)
MCZ_38282	*Manis javanica*	Outside of current range but inside the range of *M.pentadactyla*. Unclear if this is a taxonomic error or range shift.
NHMUK_98.10.5.48	*Manis javanica*	Zoo animal (Thailand)
MHNG_MHNG-MAM-1183.049	*Manis javanica*	Tanjung Gelang is the port so likely a shipping location.
PSM_Mammal-09256	*Manis pentadactyla*	Zoo animal (Honduras)
ISU_431	*Manis pentadactyla*	Zoo animal (Bloomington, USA)
UMMZ_113318	*Manis pentadactyla*	Zoo animal (Detroit, USA)
UMMZ_113316	*Manis pentadactyla*	Zoo animal (Detroit, USA)
UMMZ_113317	*Manis pentadactyla*	Zoo animal (Detroit, USA)
NHMUK_32.3.3.9	*Manis pentadactyla*	Zoo animal (India)
NHMUK_12.12.3.3	*Phataginus tricuspis*	Lindi is a port on the coast of Tanzania outside of the species range.

NHMUK_no reg 9	*Phataginus tricuspis*	Mombasa is a port on the coast of Kenya outside of the species range.
NHMUK_87.12.1.103	*Phataginus tricuspis*	Locality is Sudan, but no current pangolin ranges do not go this far north.
NHMUK_7.12.1.102	*Phataginus tricuspis*	Locality is Sudan, but no current pangolin ranges do not go this far north.
BOUM_11.Man.1	*Phataginus tricuspis*	This genus is not found in southern Africa, so locality or taxonomy must be incorrect.
MVZ_MVZ:Mamm:4829	*Phataginus tricuspis*	This genus is not found in southern Africa, so locality or taxonomy must be incorrect.
AMNH_M-216259	*Phataginus tricuspis*	This genus is not found in southern Africa, so locality or taxonomy must be incorrect.
SUI_18440	*Phataginus tricuspis*	This genus is not found in southern Africa, so locality or taxonomy must be incorrect.
O_18156	*Phataginus tricuspis*	This genus is not found in southern Africa, so locality or taxonomy must be incorrect.
GNM_Ma ex 1292	*Phataginus tricuspis*	This genus is not found in southern Africa, so locality or taxonomy must be incorrect.
GNM_CollAn 7491	*Phataginus tricuspis*	This genus is not found in southern Africa, so locality or taxonomy must be incorrect.
MNHN_MO-1869-43	*Phataginus tricuspis*	Zoo animal (Gold Coast, Australia)
UMMZ_156537	*Phataginus tricuspis*	This genus is not found in Asia, so locality or taxonomy must be incorrect.
MNHN_MO-1899-647	*Phataginus tricuspis*	This genus is not found in Asia, so locality or taxonomy must be incorrect.

Table S2: Specimen records modified after georeferencing. There are two types of edit; ***Taxonomy****, where we corrected the taxonomy of specimens with localities more than 100km from the current species range and unambiguously within the range of another closely related species; and* ***Coordinates****, where we corrected incorrect GBIF coordinates. This does not include specimens with extents > 1000km as these are automatically removed from the analyses.*

Specimen	Species	Edit	Justification
RBINS_301B	*Manis crassicaudata*	Taxonomy	Recorded in GBIF as *M. pentadactyla* but this species does not occur in Sri Lanka so must be *M. crassicaudata.*
ROM_2603230109	*Manis crassicaudata*	Taxonomy	Recorded in GBIF as *M. pentadactyla* but this species does not occur in India so must be *M. crassicaudata.*
NHMUK_77.3.14.8	*Manis crassicaudata*	Taxonomy	Recorded in GBIF as *M. pentadactyla* but this species does not occur in Sri Lanka so must be *M. crassicaudata.*
NHMUK_91.1.31.1	*Manis crassicaudata*	Taxonomy	Recorded in GBIF as *M. pentadactyla* but this species does not occur in India so must be *M. crassicaudata.*

FMNH_62918	*Manis culionensis*	Coordinates	GBIF coordinates are in the ocean.
AMNH_M-242095	*Manis culionensis*	Coordinates	GBIF coordinates are in the ocean.
KU_165510	*Manis culionensis*	Coordinates	GBIF coordinates were for the wrong locality.
FMNH_62921	*Manis culionensis*	Coordinates	GBIF coordinates were for the wrong locality.
MZLU_L897/3277	*Manis javanica*	Taxonomy	Recorded in GBIF as *M. pentadactyla* but this species does not occur in Indonesia so must be *M. javanica.*
AMNH_M-102180	*Manis javanica*	Coordinates	GBIF coordinates were for the wrong locality.
NHMUK_22.12.17.248	*Smutsia gigantea*	Taxonomy	Recorded in GBIF as *S.temminckii* but this species does not occur in West Africa so must be *S.gigantea*
MNHN_AC-VI-253	*Smutsia gigantea*	Taxonomy	Recorded in GBIF as *S.temminckii* but this species does not occur in West Africa so must be *S.gigantea*

MHNG_MHNG-MAM-601.080	*Smutsia gigantea*	Taxonomy	Recorded in GBIF as *S.temminckii* but this species does not occur in West Africa so must be *S.gigantea*
MHNG_MHNG-MAM-601.074	*Smutsia gigantea*	Taxonomy	Recorded in GBIF as *S.temminckii* but this species does not occur in West Africa so must be *S.gigantea*
O_18155	*Smutsia gigantea*	Taxonomy	Recorded in GBIF as *S.temminckii* but this species does not occur in West Africa so must be *S.gigantea*

Table S3: Percentage locality overlaps and mean percentage area overlaps for various subdivisions of the data. ***All data*** *includes all specimens except those with certainty scores of zero.* ***High quality data*** *includes only specimens with certainty scores ≥ 50% and extents < 50km.* ***No duplicates data*** *excludes duplicates, i.e. multiple specimens of the same species collected at exactly the same locality.*

All data (n = 676)				
**Species**	**Number specimens**	**% locality overlaps**	**Mean % area overlap**	**SE % area overlap**
Manis crassicaudata	23	65.22	16.85	5.11
Manis culionensis	11	90.91	11.10	2.66
Manis javanica	150	58.00	12.92	1.53
Manis pentadactyla	136	53.68	10.98	1.79
Phataginus tetradactyla	50	64.00	27.87	4.90
Phataginus tricuspis	239	67.36	31.36	2.27
Smutsia gigantea	34	50.00	17.50	5.33
Smutsia temminckii	33	42.42	28.56	6.54
**Continent**	**Number specimens**	**% locality overlap**	**Mean % area overlap**	**SE % area overlap**
Africa	359	66.40	29.04	1.84
Asia	317	56.25	12.43	1.12
**Ecology**	**Number specimens**	**% locality overlap**	**Mean % area overlap**	**SE % area overlap**
Arboreal/semi-arboreal	450	64.73	24.33	1.47
Terrestrial	226	53.19	15.13	1.76

**IUCN Red List status**	**Number specimens**	**% locality overlap**	**Mean % area overlap**	**SE % area overlap**
VU	83	57.45	28.14	3.91
EN	296	67.00	28.64	1.99
CR	297	56.15	11.97	1.13
		No duplicates (n = 362)		
**Species**	**Number specimens**	**% locality overlap**	**Mean % area overlap**	**SE % area overlap**
Manis crassicaudata	18	66.67	18.53	6.33
Manis culionensis	8	87.50	9.98	3.02
Manis javanica	79	65.82	15.77	2.63
Manis pentadactyla	59	59.32	15.35	3.66
Phataginus tetradactyla	33	75.76	33.96	6.07
Phataginus tricuspis	114	72.81	38.25	3.52
Smutsia gigantea	26	57.69	18.51	5.98
Smutsia temminckii	25	48.00	30.60	7.37
**Continent**	**Number specimens**	**% locality overlap**	**Mean % area overlap**	**SE % area overlap**
Africa	199	71.32	33.81	2.59
Asia	163	63.73	15.73	1.96
**Ecology**	**Number specimens**	**% locality overlap**	**Mean % area overlap**	**SE % area overlap**
Arboreal/semi-arboreal	234	70.87	29.09	2.22
Terrestrial	128	58.19	19.42	2.70

IUCN Red List status	Number specimens	% locality overlap	Mean % area overlap	SE % area overlap
VU	58	65.64	32.51	4.66
EN	158	71.93	32.76	2.89
CR	146	63.65	15.28	2.05

High quality data (n = 269)

Species	Number specimens	% locality overlap	Mean % area overlap	SE % area overlap
Manis crassicaudata	12	91.67	24.80	8.81
Manis culionensis	9	100.0	12.10	2.99
Manis javanica	48	100.0	25.14	3.36
Manis pentadactyla	68	98.53	20.33	2.95
Phataginus tetradactyla	17	100.0	40.91	8.64
Phataginus tricuspis	97	100.0	47.67	3.05
Smutsia gigantea	11	100.0	45.72	12.27
Smutsia temminckii	7	100.0	62.00	5.10

Continent	Number specimens	% locality overlap	Mean % area overlap	SE % area overlap
Africa	132	100.0	47.40	2.71
Asia	137	98.88	21.87	2.04

Ecology	Number specimens	% locality overlap	Mean % area overlap	SE % area overlap
Arboreal/semi-arboreal	171	100.0	38.80	2.32
Terrestrial	98	98.38	26.70	2.95

IUCN Red List status	Number specimens	% locality overlap	Mean % area overlap	SE % area overlap
VU	24	100.0	47.06	6.54
EN	120	99.88	45.20	2.88
CR	125	99.03	21.58	2.08
No duplicates, high quality data (n = 162)				
**Species**	**Number specimens**	**% locality overlap**	**Mean % area overlap**	**SE % area overlap**
Manis crassicaudata	11	90.91	26.81	940
Manis culionensis	6	100.0	11.11	3.63
Manis javanica	37	100.0	26.14	4.33
Manis pentadactyla	30	96.67	26.47	6.01
Phataginus tetradactyla	13	100.0	43.37	9.72
Phataginus tricuspis	49	100.0	54.11	4.36
Smutsia gigantea	9	100.0	43.23	13.19
Smutsia temminckii	7	100.0	62.00	5.10
**Continent**	**Number specimens**	**% locality overlap**	**Mean % area overlap**	**SE % area overlap**
Africa	78	100.0	51.77	3.55
Asia	84	98.31	25.27	3.12
**Ecology**	**Number specimens**	**% locality overlap**	**Mean % area overlap**	**SE % area overlap**
Arboreal/semi-arboreal	105	100.00	40.47	3.13
Terrestrial	57	96.44	33.54	4.45

IUCN Red List status	Number specimens	% locality overlap	Mean % area overlap	SE % area overlap
VU	20	100.0	49.89	6.77
EN	69	99.58	94.76	3.97
CR	73	98.70	25.04	3.32

Table S4: Results of beta regression models investigating correlates between percentage area overlaps for each specimen, and collection year; continent; ecology, IUCN Red List status and species. The analyses use specimens with certainty scores ≥ 50%, extents < 50km and excluding duplicates and specimen MVZ_MVZ:Mamm:125554 (n = 161). Type refers to whether the best fitting model was one with fixed or variable precision (ϕ) for each group, and the reported ϕ values are those for the best fitting model, i.e. overall ϕ for fixed and ϕ for each group for variable ϕ models.

**predictor/group**	**type**	**mean**	**se**	**z**	**p**	**ϕ**	**ϕ se**	**ϕ z**	**ϕ p**	**pseudo r^2**
**Species**										
Manis crassicaudata	fixed	-0.737	0.354	-2.083	0.037	1.512	0.141	10.71	<0.001	0.148
Manis culionesis		-0.466	0.585	-0.797	0.426					
Manis javanica		0.163	0.401	0.406	0.685					
Manis pentadactyla		0.116	0.412	0.282	0.778					
Phataginus tetradactyla		0.827	0.482	1.715	0.086					
Phataginus tricuspis		1.065	0.394	2.705	0.007					
Smutsia gigantea		0.887	0.530	1.675	0.094					
Smutsia temminckii		1.008	0.570	1.769	0.077					
**Year**	fixed	0.008	0.004	2.905	0.004	1.389	0.133	10.45	<0.001	0.058
**Continent**										
Africa	variable	0.258	0.136	1.897	0.058	0.375	0.131	2.866	0.004	0.134
Asia		-0.926	0.192	-4.817	<0.001	0.052	0.186	0.279	0.780	
**Ecology**										
Arboreal/semi-arboreal	variable	-0.116	0.118	-0.984	0.325	0.320	0.111	2.877	0.004	0.013
Terrestrial		-0.253	0.204	-1.238	0.216	-0.144	0.188	-0.765	0.444	
**IUCN**										
VU	variable	0.159	0.262	0.606	0.545	0.463	0.260	1.784	0.075	0.070
EN		-0.0387	0.300	-0.126	0.900	-0.187	0.293	-0.638	0.523	
CR		-0.810	0.300	-2.703	0.007	-0.049	0.296	-0.167	0.868	

Table S5: Results of beta regression models investigating correlations between percentage area overlaps for each specimen, and changes in human population size or land-use since 1850, 1900 or 1950. Human population size variables are log population count (popc) and log population density (popd). Land-use types are forested primary land (primf), non-forested primary land (primn) and urban land (urban). The analyses use specimens with certainty scores ≥ 50%, extents < 50km and excluding duplicates and specimen MVZ_MVZ:Mamm:125554 (n = 161). Type refers to whether the best fitting model was one with fixed or variable precision (ϕ) and the reported ϕ values are those for the best fitting model.

predictor/ time bin	type	mean	se	z	p	ϕ	ϕ se	ϕ z	ϕ p	pseudo r^2
**popc**										
1850	variable	-0.075	0.035	-2.118	0.034	0.051	0.032	1.570	0.116	0.027
1900	variable	-0.074	0.035	-2.109	0.035	0.050	0.032	1.561	0.119	0.028
1950	variable	-0.070	0.036	-1.940	0.052	0.051	0.033	1.543	0.123	0.021
**popd**										
1850	variable	-0.0094	0.040	-2.363	0.018	0.059	0.037	1.583	0.113	0.032
1900	variable	-0.094	0.040	-2.350	0.029	0.059	0.037	1.577	0.115	0.032
1950	variable	-0.091	0.041	-2.227	0.026	0.059	0.038	1.556	0.120	0.028
**primf**										
1850	fixed	1.261	0.124	0.363	0.716	1.368	0.125	10.96	< 0.001	0.065
1900	fixed	1.349	0.123	2.815	0.005	1.352	0.123	11.00	< 0.001	0.052
1950	fixed	1.681	0.763	2.202	0.028	1,327	0.120	11.06	< 0.001	0.033
**primnc**										
1850	fixed	-0.122	0.533	-0.228	0.820	1.287	0.115	11.15	<0.01	<0.001
1900	fixed	-0.109	0.553	-0.197	0.844	1.287	0.115	11.15	< 0.001	<0.001
1950	variable	0.216	0.663	0.326	0.745	-1.605	0.743	-2.161	0.031	<0.001
**urban**										
1850	variable	-0.309	0.815	-0.380	0.704	-0.644	0.706	-0.911	0.362	<0.001
1900	variable	-0.470	0.945	-0.497	0.619	-0.707	0.826	-0.856	0.392	0.001
1950	variable	-0.864	1.300	-0.664	0.506	-0.745	1.160	-0.642	0.521	0.002
